# Case Report: Van Wyk–Grumbach syndrome presenting as vaginal bleeding: diagnostic value of pituitary and pelvic imaging

**DOI:** 10.3389/fped.2025.1736671

**Published:** 2026-01-12

**Authors:** Yuqing Cao, Xiaorui Zhao, Wenjing Huang, Yue Xi, Huiying Wu

**Affiliations:** Department of Radiology, Guangzhou Women and Children’s Medical Center, Guangzhou Medical University, Guangzhou, Guangdong, China

**Keywords:** children, hypothyroidism, magnetic resonance imaging, pituitary hyperplasia, precocious puberty, Van Wyk–Grumbach syndrome

## Abstract

**Background:**

Van Wyk–Grumbach syndrome (VWGS) is a rare endocrine disorder caused by long-standing severe primary hypothyroidism, characterized by pseudoprecocious puberty and polycystic ovarian lesions. Owing to its overlapping features with central precocious puberty and ovarian tumors, misdiagnosis is common and may lead to unnecessary surgical intervention or delayed treatment.

**Case presentation:**

We report the case of an 8-year-old girl who presented with vaginal bleeding. Physical examination revealed partial precocious puberty (Tanner stage 3). Laboratory evaluation demonstrated markedly elevated thyroid-stimulating hormone (TSH >150 mIU/mL) and decreased thyroid hormones, consistent with primary hypothyroidism. Imaging revealed symmetrical pituitary hyperplasia (vertical diameter ≈17.7 mm) and large bilateral multilocular ovarian cysts.

**Conclusion:**

This case emphasizes the necessity of routine thyroid function screening in the differential diagnosis of pediatric precocious puberty WITH POOR LINEAR GROWTH and ovarian cysts. The coexistence of pituitary hyperplasia, bilateral ovarian cysts, and severe biochemical hypothyroidism represents the diagnostic triad of VWGS. Prompt levothyroxine replacement therapy rapidly reversed clinical manifestations and imaging abnormalities, preventing unnecessary surgery. These findings provide key diagnostic insights for pediatric endocrinologists and radiologists.

## 1 Introduction

**Figure 1:**
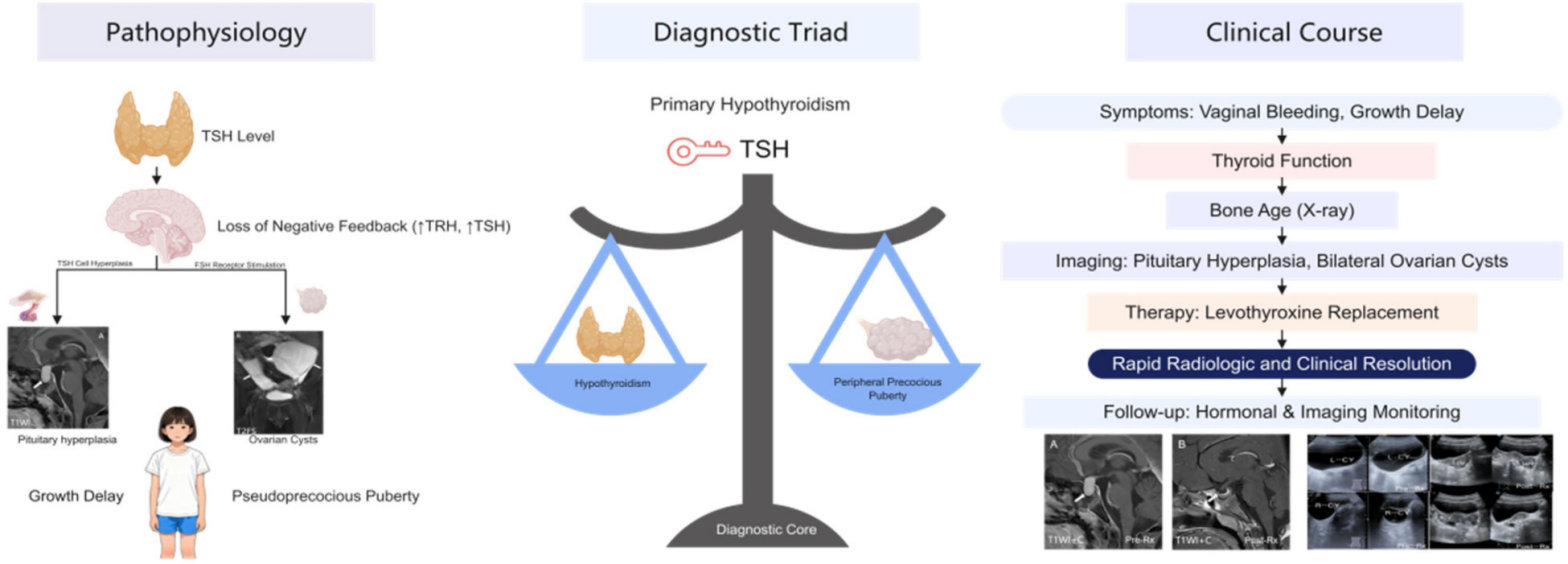
Graphical summary of Van Wyk-Grumbach syndrome (vWGS). The schematic illustrates the pathophysiologic mechanism of VWGS due to primary hypothyroidism (left), the diagnostic triad of pituitary hyperplasia, bilateral ovarian cysts, and severe hypothyroidism (center), and the clinical course with levothyroxine-induced radiologic and clinical resolution (right).

(Figure 1): Van Wyk–Grumbach syndrome (VWGS) is an uncommon endocrine disorder secondary to long-standing primary hypothyroidism. It is defined by the clinical triad of pseudoprecocious puberty, polycystic ovarian lesions, and pituitary hyperplasia (1). Since its first description in 1960, VWGS has remained an exceedingly rare etiology among pediatric endocrine conditions (1). Clinically, it combines the metabolic sluggishness of hypothyroidism with the paradoxical features of precocious sexual development, resulting in diagnostic confusion. Furthermore, hypothyroidism-induced ovarian cysts may cause an increase in tumor marker levels (2). Consequently, patients are often misdiagnosed with central precocious puberty or ovarian neoplasms, which can lead to unnecessary surgery and delayed endocrine therapy (3, 4).

Imaging may play an important role in early recognition and accurate differentiation of VWGS. Symmetrical pituitary hyperplasia on MRI, coupled with bilateral multilocular ovarian cysts on pelvic ultrasound or MRI, constitutes its hallmark imaging pattern. Comprehensive evaluation of both the pituitary and pelvic organs is therefore not universally required, but can be highly helpful in difficult cases.

This report presents the imaging and clinical features of a child with VWGS whose initial manifestation was vaginal bleeding. The aims are threefold: (1) to analyze the relationship between the imaging features and pathophysiologic mechanisms of VWGS; (2) to highlight the diagnostic value of integrated pituitary and pelvic imaging in distinguishing the etiology of pediatric precocious puberty; and (3) to provide radiologists and endocrinologists with a structured diagnostic approach for early recognition and optimal management of this rare entity.

## 2 Case presentation

An 8-year-old girl was referred to our adolescent gynecology clinic with a 5-day history of unexplained vaginal bleeding. The bleeding was mild but required the use of sanitary pads and was not accompanied by abdominal pain or fever. Abdominal ultrasound performed elsewhere suggested “bilateral ovarian cysts,” prompting further investigation. The patient's growth history revealed significant weight gain over the past two years with slowed linear growth (below age norms). She had no headaches, vomiting, visual disturbances, or developmental delays. Her birth history and family history were unremarkable.

### 2.1 Clinical examination

Height: 114 cm (Z-score:-3.3, percentile: <1%); Weight: 31.5 kg (Z-score: + 1.0, percentile: ≈85%). BMI:24.24 kg/m^2^, which is significantly elevated (Z-score >+3, percentile: >99.9%). Abdominal obesity with altered body shape. Facial appearance shows mild edema. Hair is thick and dense. Hairline at the nape of the neck is slightly low.Increased body hair was observed throughout the skin, most prominent on the lower limbs and back. No purplish-brown streaks were noted on the lower limbs. No café-au-lait spots were present on the body. No axillary or pubic hair was present. Vellus hair was increased on the limbs. Genital development: Tanner Stage I (prepubertal, no pubic hair, smooth skin in the pubic area consistent with childhood state), slightly darker in color (The hyperpigmentation observed on the skin was regular in morphology and was not accompanied by skin thickening or skin tags.), with a small amount of blood-stained red discharge from the vaginal opening. There was no clitoromegaly, and the vaginal mucosa did not appear estrogenized (lacking the moist, dull pink characteristic of estrogen effect). Breast development: Tanner Stage III (breasts and areolae continue to enlarge, breasts protrude from the chest wall, but the areolae and breasts are on the same plane).

### 2.2 Laboratory findings

Comprehensive laboratory evaluation revealed severe endocrine and metabolic disturbances (Table 1). Findings included primary hypothyroidism (markedly elevated TSH with suppressed FT3/FT4), positive thyroid autoantibodies, hyperprolactinemia, and dyslipidemia—collectively consistent with Hashimoto's thyroiditis and secondary metabolic dysfunction.

**Table 1:** Laboratory parameters at diagnosis and following treatment.

Category	Category/parameter	Pre-treatment results	6-month post-treatment results	Reference range	Variation trend
Thyroid function	TSH(mIU/mL)	>150.00	6.818	0.79-6.06	↓↓↓
	FT3 (pmol/L)	2.12	8.42	4.18-7.23	↑
	FT4(pmol/L)	10.90	34.47	14.42-22.22	↑↑
	TgAb(IU/mL)	143.50	113.40	<4.5	↓
Reproductive hormones	Prolactin (ng/mL)	81.08	6.46	2.8-29.2	↓↓↓
	Luteinizing hormone(IU/L)	0.02	1.56	0.02-0.18	↑↑
	Follicle-stimulating hormone(IU/L)	4.74	6.41	1.0-4.2	↑
	Estradiol (E2)(pmol/L)	60	161.72	0-73.4	↑↑
Biochemical profile	Creatinine (μmol/L)	69	Not tested	27-66	–
	Creatine kinase (U/L)	678	Not tested	45-390	–
	CK-MB(ng/mL)	6.39	Not tested	0-5	–
	Lactate dehydrogenase (U/L)	358	Not tested	126-294	–
	Total cholesterol (mmol/L)	8.45	Not tested	3.4-5.2	–
	Low-density lipoprotein (mmol/L)	5.51	Not tested	<3.37	–
Tumor markers	Carcinoembryonic antigen (CEA) (ng/mL)	0.68	Not tested	<5	–
	Alpha-fetoprotein (AFP)(ng/mL)	7.51	Not tested	0-8.1	–

### 2.3 Imaging findings

#### 2.3.1 Thyroid color Doppler ultrasound

**Figure 2:**
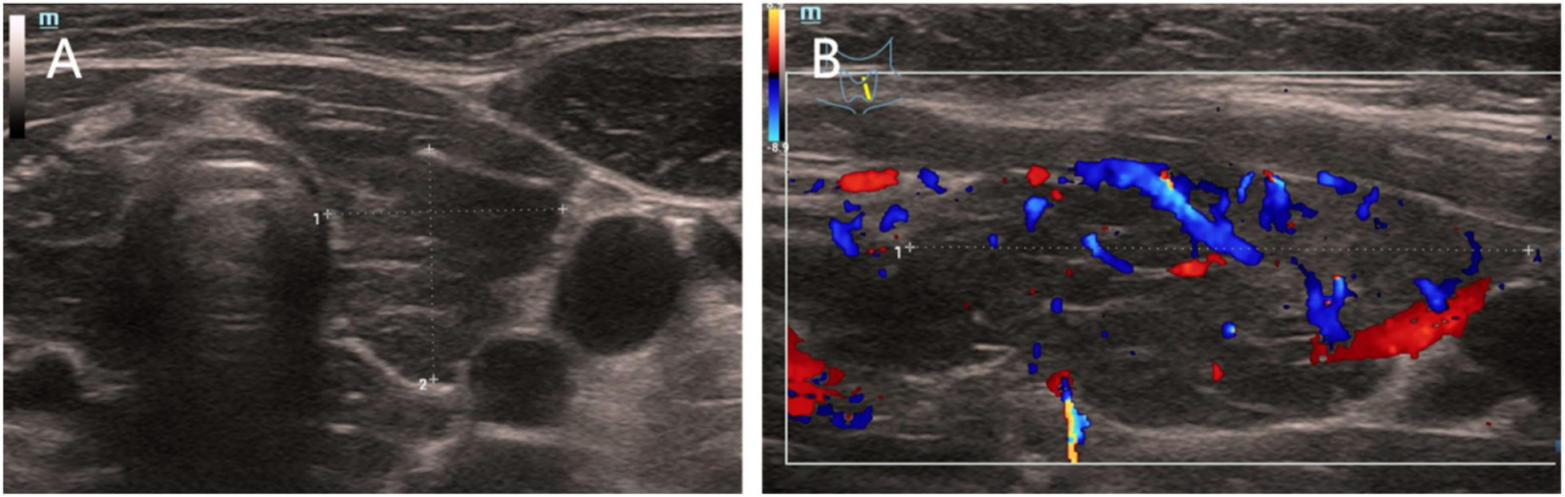
Thyroid color Doppler ultrasound. **(A)** Diffuse enlargement of the right thyroid lobe with coarse echotexture.The right thyroid lobe measured 1.39 cm × 1.36 cm. **(B)** Increased intraglandular vascularity consistent with Hashimoto's thyroiditis (C-TIRADS Class 1).

(Figure 2): Diffuse thyroid enlargement with increased vascularity was observed. The findings (C-TIRADS 1) were compatible with Hashimoto's thyroiditis. A reactive lymph node was noted in the left cervical T-zone.

#### 2.3.2 Pituitary MRI

**Figure 3:**
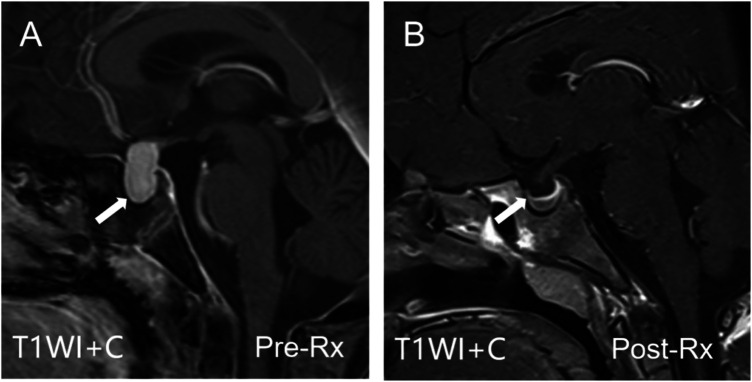
Pituitary MRI with contrast enhancement. **(A)** Sagittal contrast-enhanced T1-weighted image showing marked symmetrical pituitary enlargement (white arrow) with upward convexity and a characteristic “waist sign,” indicative of pituitary hyperplasia. **(B)** Follow-up after 4 weeks of levothyroxine therapy demonstrating significant reduction in pituitary size and near-complete restoration of normal morphology.

(Figure 3): Pre-treatment MRI revealed marked, symmetrical pituitary enlargement with upward bulging and the classic “waist sign.” The gland measured approximately 17.7 mm vertically and 15.1 mm transversely, showing homogeneous enhancement post-contrast. Follow-up MRI after 4 weeks of levothyroxine therapy demonstrated significant reduction in pituitary size with restoration of near-normal morphology.

#### 2.3.3 Pelvic MRI

**Figure 4:**
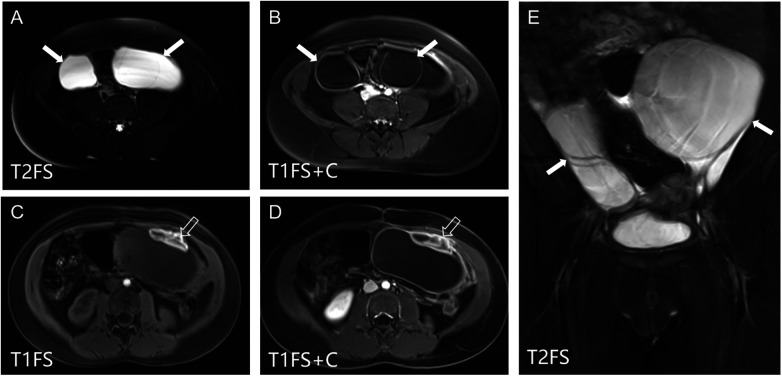
Pelvic MRI showing bilateral ovarian cysts. **(A,B,E)** Coronal and axial T1WI and T2WI reveal large multilocular cystic lesions in both adnexal regions (white arrows) with thin internal septations and well-defined borders. **(C,D)** Hemorrhagic foci within the left adnexal cyst are demonstrated by peripheral high signal intensity on T1-weighted images (hollow white arrow).

(Figure 4): Large multilocular cystic masses were observed bilaterally in the adnexal regions. The left cyst measured approximately 10.7 × 6.0 × 9.7 cm and the right cyst 4.4 × 4.3 × 8.8 cm. The cysts had thin walls and fine internal septations. Hemorrhagic foci were present in the left lesion, showing peripheral high signal intensity on T1WI.

#### 2.3.4 Transabdominal gynecological ultrasound

**Figure 5:**
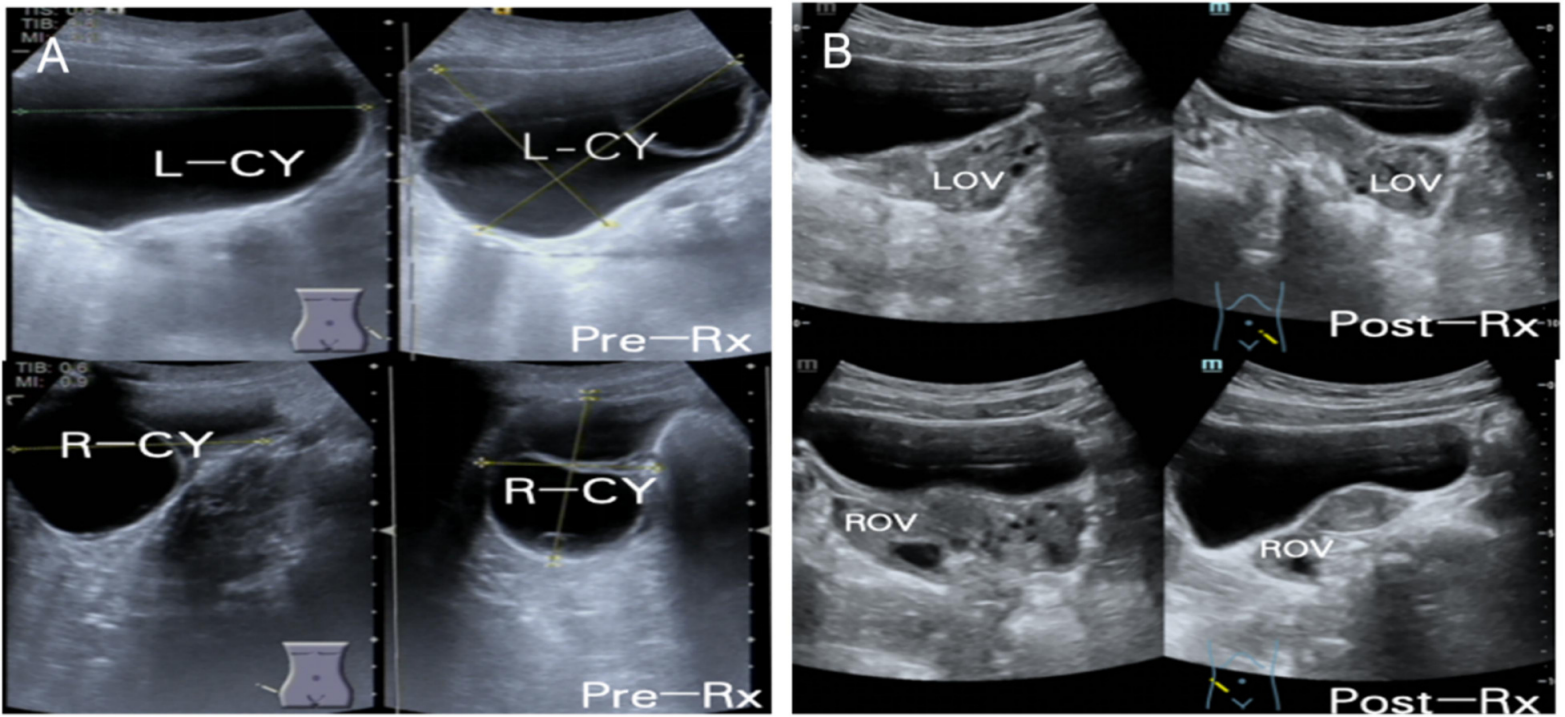
Transabdominal gynecological ultrasound before and after therapy. **(A)** Pre-treatment scan showing bilateral polycystic ovarian cysts (right: 61 × 61 × 42 mm; left: 98 × 93 × 71 mm) with residual ovarian tissue visible peripherally. **(B)** Follow-up scan obtained 4 weeks after levothyroxine therapy showing marked reduction of cystic lesions and return to normal ovarian morphology.

(Figure 5): The uterus was of normal size and shape (41 × 26 × 23 mm) with uniform myometrial echogenicity and endometrial thickness of 6.2 mm. Bilateral adnexal cystic masses were seen: right, 61 × 61 × 42 mm; left, 98 × 93 × 71 mm, with residual ovarian tissue visible at the periphery. Follow-up 4 weeks post-treatment revealed marked regression of cysts to near-normal ovarian dimensions.

#### 2.3.5 Routine bone age radiograph

**Figure 6:**
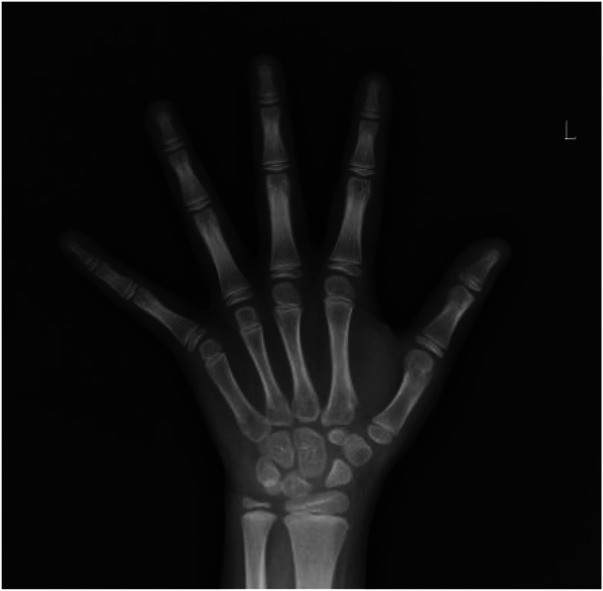
Routine bone age radiograph (bone age assessment at 4 weeks post-treatment indicates bone age equivalent to approximately 10 years of age in females).

(Figure 6): A bone age study was conducted, revealing no significant delay compared to chronological age. A bone age assessment conducted 4 weeks after treatment indicated a bone age equivalent to approximately 10 years of age in females.

## 3 Diagnosis and treatment

Final Diagnosis: (1) Van Wyk-Grumbach syndrome; (2) Primary hypothyroidism secondary to Hashimoto's thyroiditis. The patient was initiated on levothyroxine replacement therapy at a starting dose of 25 mcg per day (approximately 0.8 mcg/kg/day). The dose was gradually titrated over a 4-week period to a maintenance dose of 62.5 mcg per day (approximately 2.0 mcg/kg/day). Clinical symptoms improved significantly, and follow-up imaging confirmed regression of pituitary and ovarian abnormalities (Figures 3B, 5B).

## 4 Discussion

Van Wyk-Grumbach syndrome (VWGS) typically manifests with pseudoprecocious puberty and delayed bone age in the setting of severe hypothyroidism. Although our patient exhibited classic signs such as growth retardation and polycystic ovaries (5), the bone age was not delayed—a finding inconsistent with classic VWGS. This discrepancy, along with post-treatment hormonal evidence of central activation (elevated LH and estradiol), justified comprehensive imaging. Pelvic MRI aided the initial assessment, while pituitary MRI revealed the pituitary vertical diameter (17.7 mm) exceeded the mean (15.5 ± 2.1 mm) reported by Rathod et al. (1), likely reflecting the patient's extremely elevated TSH level (>150 mIU/mL). Given the advanced bone age and emergence of central puberty signs, the follow-up MRI was primarily performed to exclude organic pathology, thereby supporting the diagnosis of a unique VWGS variant that subsequently evolved into physiologic central puberty. The imaging findings confirmed the absence of other lesions, providing important evidence for this distinctive clinical presentation.

### 4.1 Pathophysiology

The pathogenesis of VWGS is incompletely understood. The most accepted theory involves severe thyroid hormone deficiency, which removes negative feedback inhibition on the hypothalamic–pituitar*y* axis (1). This results in sustained thyrotropin-releasing hormone (TRH) stimulation, causing both pituitary thyrotroph hyperplasia and, through molecular mimicry, cross-activation of follicle-stimulating hormone (FSH) receptors. Consequently, ovarian follicular stimulation and estrogen secretion occur despite suppressed gonadotropins, leading to pseudoprecocious puberty (6).

However, the present case demonstrates several important deviations from the typical VWGS presentation. While the patient initially exhibited classic FSH predominance and ovarian cysts consistent with thyrotropin-mediated gonadotropin cross-reactivity, the non-delayed bone age—an unusual finding in this context—suggested that the inhibitory effect of profound hypothyroidism on the hypothalamic-pituitary-gonadal (HPG) axis (which normally suppresses bone maturation and central puberty) was either incomplete or of shorter duration.

Following rapid correction of hypothyroidism, the removal of this central inhibition-potentially mediated by elevated TRH and subsequent suppression of gonadotropin-releasing hormone (GnRH) pulsatility-may have triggered the activation of true central puberty. This is evidenced by the rise in LH and sustained elevation of estradiol levels at the 6-month follow-up. This “unmasking” or disinhibition of the central HPG axis could explain the convergence of clinical features: an initial FSH-predominant state consistent with VWGS, followed by progression into true, albeit age-appropriate, central puberty, accounting for the advanced breast development.

### 4.2 The core value of imaging in diagnosis and differential diagnosis

Imaging may be helpful in the diagnosis and differential diagnosis of VWGS in some cases.

#### 4.2.1 Pituitary evaluation

MRI typically shows symmetrical enlargement with homogeneous enhancement and the “waist sign,” differentiating VWGS from pituitary adenomas, which are often asymmetric and heterogeneously enhancing. Correct identification prevents unnecessary surgical intervention.

#### 4.2.2 Pelvic evaluation

Pelvic evaluation followed a rational diagnostic pathway. Initial ultrasound revealed bilateral giant cystic masses with heterogeneous echogenicity and septal thickening. While suggestive of VWGS, the size and complexity warranted MRI to exclude neoplasms. MRI confirmed bilateral, thin-walled, multilocular cysts without solid components or enhancement, with left cyst hemorrhage (T1WI high signal)—features distinguishing VWGS from tumors or simple cysts. This imaging combination helped exclude differential diagnoses like granulosa cell tumors. In any child presenting with bilateral ovarian cysts, VWGS should be strongly suspected (7).

#### 4.2.3 Multimodal integration

Simultaneous pituitary and pelvic imaging provides complementary diagnostic evidence, reinforcing the importance of a multidisciplinary imaging approach in complex pediatric endocrine conditions.

### 4.3 Clinical and educational implications

VWGS is entirely reversible with appropriate thyroid hormone replacement. This case underscores the necessity of including thyroid function testing in the evaluation of children with precocious puberty or ovarian cysts-particularly those with growth retardation or other hypothyroid features (4). Pituitary and pelvic MRI can serve as valuable diagnostic adjuncts in specific cases, facilitating early recognition and avoidance of unnecessary procedures. Pituitary MRI may be useful for confirming central etiology, while pelvic MRI may be considered when ultrasound fails to characterize adnexal lesions definitively; together they aid in differential diagnosis and help avoid unnecessary interventions.

Beyond clinical management, this case holds substantial educational value for radiologists and endocrinologists, demonstrating how precise imaging interpretation can guide targeted, noninvasive therapy. The rapid normalization of imaging and hormonal findings following levothyroxine therapy highlights the condition's excellent prognosis when accurately diagnosed.

## Data Availability

The original contributions presented in the study are included in the article/Supplementary Material, further inquiries can be directed to the corresponding author.
